# Investigation of corticosteroid levels in the hairs of female Holstein calves

**DOI:** 10.1111/asj.70016

**Published:** 2024-12-04

**Authors:** Hiroki Fushuku, Nobuyoshi Matsunaga

**Affiliations:** ^1^ Department of Life and Food Sciences, School of Agriculture and Animal Science Obihiro University of Agriculture and Veterinary Medicine Obihiro Japan

**Keywords:** female Holstein calves, hair color and region, hair corticoid, season

## Abstract

This study was designed to investigate the degree of long‐term effects by measuring cortisol and aldosterone concentrations in the growing hair including lipid which is absorbed from the blood of six female Holstein calves by enzyme‐linked immunosorbent assay (ELISA). The total number of calves used was 18 (three seasons). Three comparative factors were used: region (back and chest), hair color (black and white), and season (summer, winter, and spring). The hair cortisol of the back region (white color) was higher than the other region and color (*p* < 0.05). The measured value in the spring season (back region) was significantly higher than that in the summer (chest region) (*p* < 0.05). Although there has been no report on the hair aldosterone until now, it was possible to measure the concentration in the present study. The hair aldosterone of the back region was higher than the chest region (*p* < 0.01), and the white hair color was higher than the black hair color (*p* < 0.05). The measured value in the spring season was also significantly higher than that in the summer season and the winter season (*p* < 0.01). This result suggested the changes are affected by the hair region factor, the hair color factor, and the changing meteorological conditions.

## 1 INTRODUCTION

Cortisol is a type of glucocorticoid, which is produced in the adrenal cortex and has functions such as the regulating lipid, protein metabolism, and the increasing blood glucose concentration. This is a main glucocorticoid that shows the character of increase by stress. Previous studies have been conducted on the other animals (Heimburge et al., 2019). In an example using rhesus monkeys, the cortisol concentration in the high‐density environment troop was higher than that in the low‐density environment. In addition, the cortisol concentration of the individuals with lower ranks within the troop was higher than that of the individuals with higher ranks (Dettmer et al., 2014). In salmon‐feeding grizzly bears, a significant negative correlation was found between the cortisol concentrations and the salmon availability (Bryan et al., 2013). In an example using sheep, the cortisol concentration in the hair of sheep restricted from drinking water for 3 h after feeding was higher than that of the sheep allowed to drink freely. The availability of water was an indicator of stress (Nejad et al., 2014). In horses, the newborn foals were higher than the cortisol concentrations of 30‐ or 60‐day‐old foals (Comin et al., 2012; Montillo et al., 2014). The studies in American black bears (Lafferty et al., 2015) and coyotes (Schell et al., 2017) showed that the hair cortisol concentrations in the male was higher than that in the female (Bechshoft et al., 2011). The opposite result was found for polar bears (Cattet et al., 2014) and brown bears (Kapoor et al., 2018). The above research examples clearly demonstrate that the age differences, the sex differences, and the environment within the group influence the cortisol concentration in the hair. There are some examples of studies using cattle. As an example of previous research, the presence or absence of surgical castration was a factor of significant changes in the hair cortisol concentrations (Gonzalez et al., 2010; Petherick et al., 2014). In addition, Uetake et al. investigated the effects of calving number, lactation period, and month on the hair cortisol concentration using lactation cows in a cold‐ and a warm‐temperate region out of four climatic zones in Japan (Uetake et al., 2018). On the other hand, aldosterone is a type of mineralocorticoid that is produced in the adrenal cortex and increases the blood pressure by promoting the sodium reabsorption in the animal's kidneys. A study measured aldosterone in the hair of polar bears using high‐performance liquid chromatography (HPLC) and enzyme‐linked immunosorbent assay (ELISA) that can quantify multiple steroids (Weisser et al., 2016). However, while the cortisol concentrations can measure to investigate regional differences in the hair, the aldosterone concentrations in the hair were below the detection limit and no useful results can be obtained. These hormones stored in the hair are relatively stable and less influenced by the circadian and ultradian rhythms of the experimental animals. In addition, the sampling is less invasive and easier to measure than the blood sample or the saliva sample collection. Therefore, this study was designed to investigate the changes in the both hormone (cortisol and aldosterone) concentrations in the growing hair including lipid which is absorbed from the blood of female Holstein calves over a long period. Three comparative factors were used: region (back and chest), hair color (black and white), and season (summer, winter, and spring).

## 2 MATERIALS AND METHODS

### 2.1 Animals

All procedures used in this experiment were approved by the Animal Care and Use Committee of the Obihiro University of Agriculture and Veterinary Medicine (Obihiro, Japan). Three trials were conducted on August 18, 2022; December 21, 2022; and April 13, 2023, with six female Holstein calves raised at Obihiro University of Agriculture and Veterinary Medicine Field Science Center. The total number of calves used was 18. The six average age were 5.3 months at the summer season (August), 5.2 months at the winter season (December), and 5.1 months at the spring season (April), respectively. The temperature during the measurement period is shown in the table below, and the measurement period for meteorological data was set the average during 2 months before the test month (Table 1). On the day of the experiment, six female Holstein calves were tethered with bridles, and a total of more than 1 g of hair was collected from the entire length of hair from the root to tip of each calf using clippers. At this time, the black color hair and the white color hair were collected from the back region and the chest region, respectively. After collecting the hair, it was stored at 4°C.

**TABLE 1:** The meteorological data of Obihiro City from Japan Meteorological Agency.

Date and time: August 18, 2022 (summer); December 21, 2022 (winter); April 13, 2023 (spring).			
	Summer	Winter	Spring
Average maximum temperature (°C)	23.6	13.5	4.3
Average minimum temperature (°C)	15.0	2.8	−7.7
Daily average temperature (°C)	18.6	7.9	−1.7

### 2.2 Extraction

Hair cortisol and aldosterone were extracted by the method of Uetake et al. with slight modification (Uetake et al., 2018). After storing in a cool (4°C) and dark place, the hair sample was transferred to a 50‐mL tube filled with distilled water and placed in an ultrasonic cleaner at 50°C for 10 min. The sample was then transferred to a 20‐mL tube to drain moisture and dried by air in a draft chamber over 48 h. After the sample was dried, it was cut into powder using scissors. In addition, 2 mL of methanol was added to 50 mg of the sample and the mixture was left at 37°C over 18 h for extraction. The methanol was then dried by air in a draft chamber, and 400 μL of assay buffer for each measurement was added. After stirring all tubes with a vortex mixer for 30 s, the separation was performed with a centrifuge at 12,000 rpm for 5 min and the supernatant was removed and stored to freeze at −20°C.

### 2.3 Cortisol assay

The concentration of cortisol was measured using a commercially available ELISA kit (DetectX Cortisol Enzyme ImmunoAssay Kit, Arbor Assays, USA). The Lot No. were 21C017c for the summer season (August), 21C025c for the winter season (December), and 21C025b for the spring season (April), respectively. The cortisol assay revealed ED_50_ of 966.7 pg/mL. Intra‐ and inter‐assay coefficients of variation were 7.3% and 10.4%, respectively.

### 2.4 Aldosterone assay

The concentration of aldosterone was measured using a commercially available ELISA kit (DetectX Aldosterone Enzyme Immunoassay Kit, Arbor Assays, USA). The Lot No. were 23AL004a for the summer season (August), 22AL009k for the winter season (December), and 22AL009h for the spring season (April), respectively. The aldosterone assay revealed ED_50_ of 416.7 pg/mL. Intra‐ and inter‐assay coefficients of variation were 6.9% and 15.5%, respectively.

### 2.5 Statistics

Mean values as well as the standard errors of the means were calculated. Each region, color, and season were statistically analyzed by three‐way analysis of variance (ANOVA) using the general linear model (GLM) procedure of the SAS program package (SAS Institute, USA) followed by post hoc test as needed. Aldosterone assay showed data below the detection limit during the summer season, with a back and black sample and three each for chest and black and chest and white samples. Therefore, these samples were excluded from the statistical analysis.

## 3 RESULTS

### 3.1 Cortisol

**FIGURE 1:**
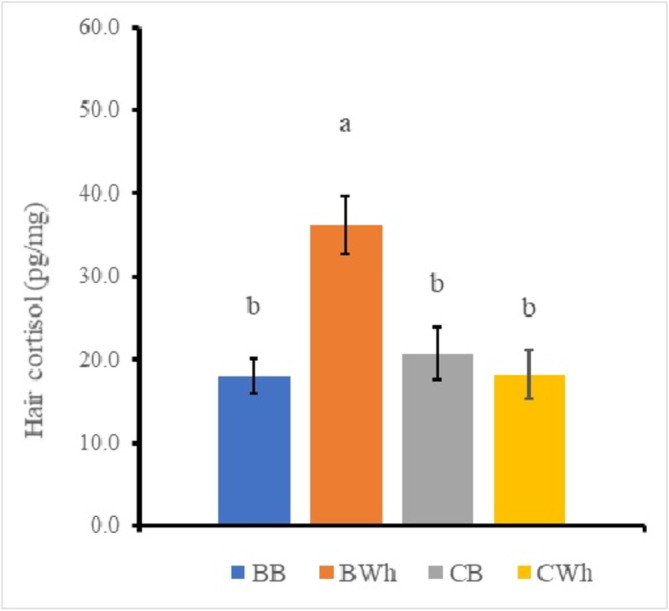
Effects of the region factor and the color factor on the hair cortisol in female Holstein calves. Mean values (in picogram per milligram) for six calves are shown: the vertical bars represent the SEM. Different colors indicate BB (back and black), BWh (back and white), CB (chest and black), and CWh (chest and white), respectively. Data for each region and color were analyzed by Tukey–Kramer multiple range analysis after ANOVA. Different characters indicate significant differences (*p* < 0.05). ANOVA, analysis of variance; SEM, standard error of the mean.

**FIGURE 2:**
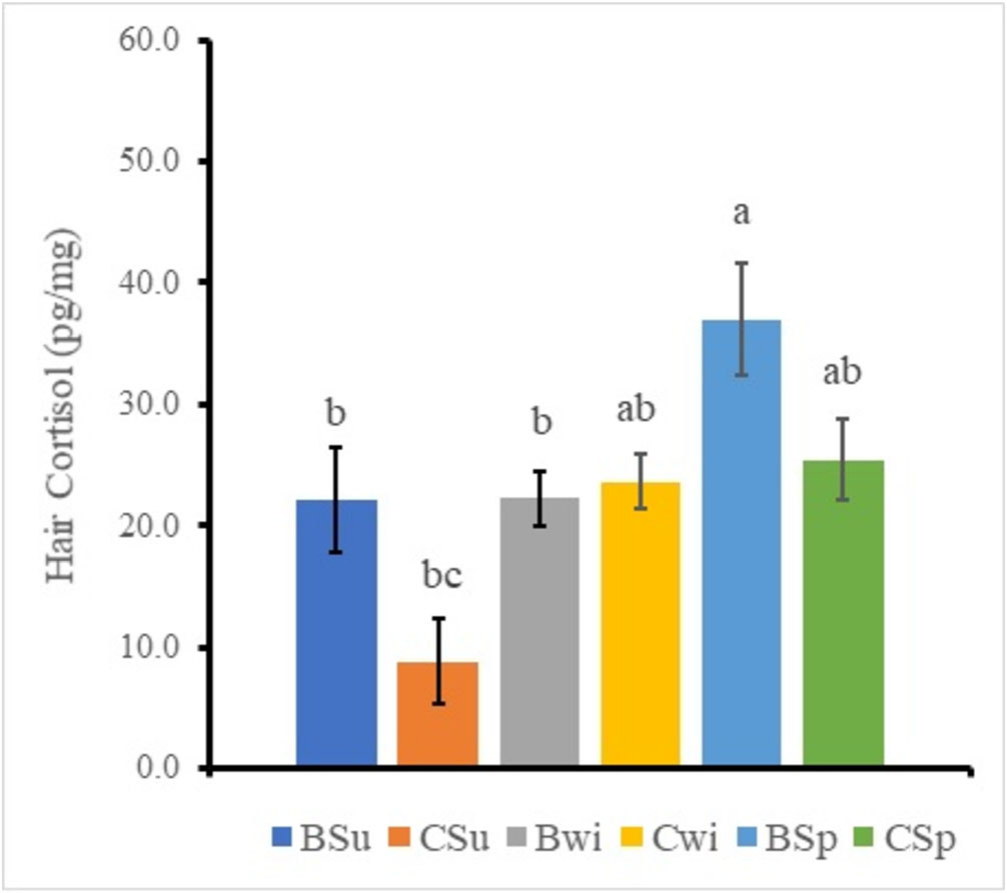
Effects of the region factor and the season factor on the hair cortisol in female Holstein calves. Mean values (in picogram per milligram) for six calves are shown: the vertical bars represent the SEM. Different colors indicate BSu (back and summer), CSu (chest and summer), BWi (back and winter), CWi (chest and winter), BSp (back and spring), and CSp (chest and spring), respectively. Data for each region and color were analyzed by Tukey–Kramer multiple range analysis after ANOVA. Different characters indicate significant differences (*p* < 0.05). ANOVA, analysis of variance; SEM, standard error of the mean.

Three‐way ANOVA for each scale on region, color, and season analyzes to clarify differences of the factors. The result identified significant main effects in the region factor and the color factor and the two‐way interaction effect (Region * Color) (Table 2). Therefore, the significant difference of the region data and the color data were analyzed by Tukey–Kramer multiple comparison procedure on the values pooled within each data classification, because there were four factors. The region and color samples showed the back and black sample (18.0 ± 2.0 pg/mg), the back and white sample (36.2 ± 3.5 pg/mg), the chest and black sample (20.8 ± 3.0 pg/mg), and the chest and white sample (18.3 ± 2.8 pg/mg), respectively (Figure 1). A synergistic effect was observed between the region factor and the color factor. The only back and white sample was significantly higher (*p* < 0.05) than the other samples (19.0 ± 1.5 pg/mg). The statistic also identified significant difference among main effects of the region factor and the season factor and the two‐way interaction effect (Region * Season) (Table 2). Therefore, the significant difference of the region data and the color data were analyzed by Tukey–Kramer multiple comparison procedure on the values pooled within each data classification, because there were six factors. The region and season samples showed the back and summer sample (22.1 ± 4.3 pg/mg), the chest and summer sample (8.9 ± 3.5 pg/mg), the back and winter sample (22.3 ± 2.3 pg/mg), the chest and winter sample (23.7 ± 2.2 pg/mg), the back and spring sample (37.0 ± 4.6 pg/mg), and the chest and spring sample (25.5 ± 3.0 pg/mg), respectively (Figure 2). A synergistic effect was observed between the region factor and the season factor. The back and spring sample was significantly higher than the back and summer sample, the chest and summer sample, and the back and winter sample, respectively (*p* < 0.05). The only chest and summer sample was significantly lower than the back and spring sample (*p* < 0.05). Region effect and season effect were recognized in this figure.

**TABLE 2:** Three‐way ANOVA results for the comparison of different region, color, and season on the hair cortisol.

Source	Type III sum of squares	df	Mean square	*F*	Pr > F
Region	1096.15	1	1096.15	12.28	0.01
Color	1216.82	1	1216.82	13.63	0.01
Season	2948.31	2	1474.15	16.52	0.01
Region * Color	1673.71	1	1673.71	18.75	0.01
Region * Season	739.91	2	369.95	4.15	0.05
Color * Season	411.94	2	205.97	2.31	n.s
Region * Color * Season	465.74	2	232.87	2.61	n.s
Error	5176.32	58	89.25	‐	‐
Corrcted total	13674.49	69	‐	‐	‐
*R* square = 0.621					

Abbreviations: ANOVA, analysis of variance; n.s., not significant.

### 3.2 Aldosterone

**FIGURE 3:**
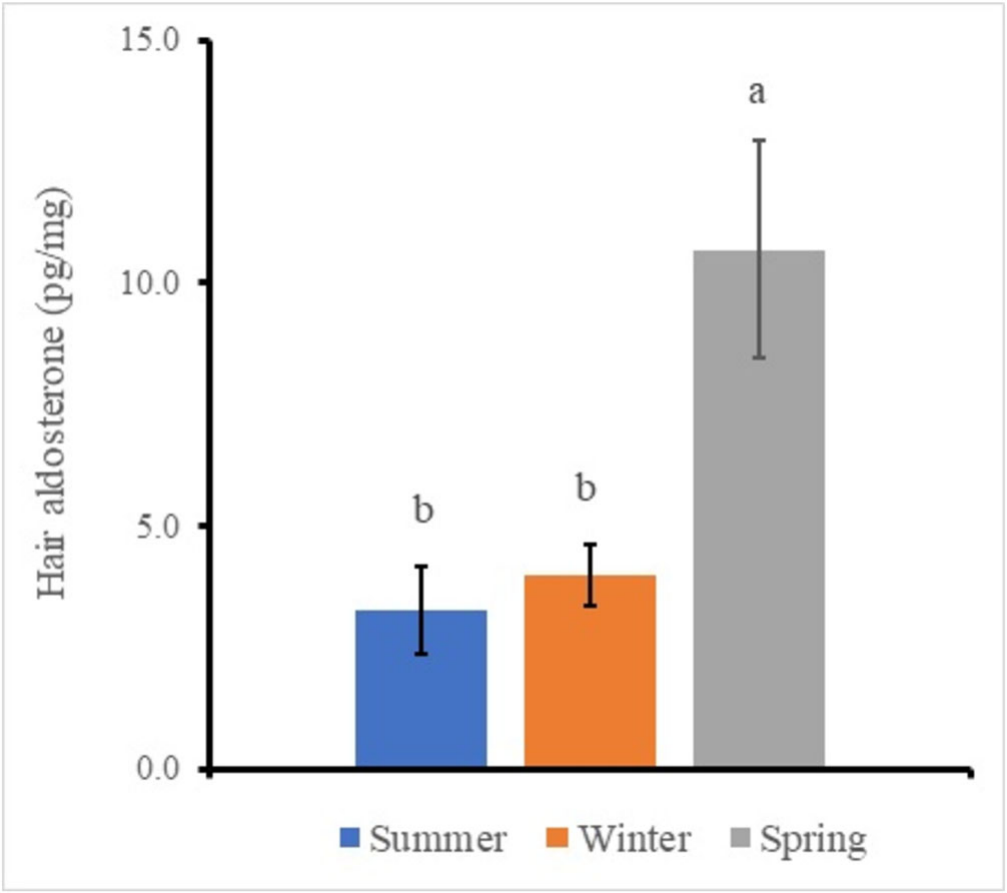
Effect of the season factor on the hair aldosterone in female Holstein calves. Mean values (in picogram per milligram) for six calves are shown: the vertical bars represent the SEM. Different colors indicate summer, winter, and spring, respectively. Data for each season was analyzed by Tukey–Kramer multiple range analysis after ANOVA. Different characters indicate significant differences (*p* < 0.05). ANOVA, analysis of variance; SEM, standard error of the mean.

Three‐way ANOVA for each scale on region, color, and season analyzes to clarify differences of the factors. The result identified significant main effects in the region factor, the color factor, and the season factor, respectively (Table 3). The region samples showed the back sample (7.4 ± 1.1 pg/mg) and the chest sample (5.1 ± 0.9 pg/mg), respectively (figure not shown), and the back sample was significantly higher than the chest sample (*p* < 0.01). The color samples showed the black sample (5.3 ± 1.2 pg/mg) and the white sample (7.4 ± 0.9 pg/mg), respectively (figure not shown), and the white sample was significantly higher than the black sample (*p* < 0.05). The significant difference of the season data was analyzed by Tukey–Kramer multiple comparison procedure on the values pooled within each data classification, because there were three factors. The season samples showed the summer sample (3.3 ± 1.1 pg/mg), the winter sample (4.0 ± 0.7 pg/mg), and the spring sample (10.7 ± 2.2 pg/mg), respectively (Figure 3). The only spring sample was significantly higher (*p* < 0.05) than the other samples (3.7 ± 0.4 pg/mg).

**TABLE 3:** Three‐way ANOVA results for the comparison of different region, color, and season on the hair aldosterone.

Source	Type III sum of squares	Df	Mean square	*F*	Pr > F
Region	142.91	1	142.91	8.26	0.01
Color	71.67	1	71.67	4.14	0.05
Season	806.24	2	403.12	23.31	0.01
Region * Color	56.74	1	56.74	3.28	n.s.
Region * Season	62.62	2	31.31	1.81	n.s.
Color * Season	44.87	2	22.43	1.30	n.s
Region * Color * Season	99.66	2	49.83	2.88	n.s
Error	882.16	51	17.30	‐	‐
Corrcted total	2121.88	62	‐	‐	‐
*R* square = 0.585					

Abbreviations: ANOVA, analysis of variance; n.s., not significant.

## 4 DISCUSSION

Previous experiment has reported that the cortisol concentrations in the hair of dairy cows was the range from 2 to 20 pg/mg (Uetake et al., 2018). The hair cortisol values were slightly lower than that of the present study. This difference of the hair cortisol concentration may cause by the age, the cortisol measurement method, and the sampling region. The measurement period for meteorological data was set the average during 2 months before the test month in the present study. Hair growth consists of three stages: active growth (anagen phase), transition (transition phase), and rest (telogen phase). Although the hair growth also changes depending on the season, it is necessary to consider time lag when substances are taken into the hair (Kapoor et al., 2018). Furthermore, considering that the hair is used as a biomarker of stress grows by an average of about 0.6 mm per day, this is thought to reflect the accumulation of hormones at least over a month. The back region and the white color sample was significantly higher than the other samples (Figure 1). Because decomposition of female hormones in treated wastewater by ultraviolet (UV) rays was reported (Matsuyama et al., 2004), the decomposition of cortisol contained a steroid hormone in the black hair as well as sex hormones may be more sensitive to UV contained in sunlight than the white color. It is also possible that the back region has less body fat storage which is absorbed steroid hormone from the blood than the chest region. These results indicate the changes during the accumulation in the hair cortisol from the blood over a long period. The measured value in the spring season (back region) was significantly higher than that in the summer season (chest region) in the present study (Figure 2). There have been many reports showing that exposure to cold increases blood cortisol level (Hiramatsu et al., 1984; Shida et al., 2020). The female Holstein calves exposed a low average temperature rearing environment of approximately under 0°C during the spring season is considered to be a cause of the cold stress. In addition, the cortisol concentration in the hair showed the low value in the summer season; the average temperature may be within the range of zone of thermoneutrality (Table 1). Therefore, it is possible that the value in the hair of the spring season was higher than that of the summer season because of the accumulation of the hair from the blood. In addition, the back region in the hair was higher than the chest region as with the region factor, the color factor, and the two‐way interaction effect (Region * Color) (Figure 1).

On the other hand, the hair aldosterone of the back region and the white hair color were significantly higher than the chest region and the black color, respectively, in the present study. Because aldosterone is a member of the steroid family and shows the same chemical characters such as UV sensitivity and lipid solubility, these changes explain due to the same reasons as for cortisol measurements. The measured value in the spring season was significantly higher than that in the other seasons in the present study (Figure 3). There have been many reports that the cold exposure stimulated the sympathetic nerves and the released blood adrenaline; the adrenaline increases the blood pressure and secretes the blood aldosterone (Hiramatsu et al., 1984). Therefore, it is possible that the value in the hair of the spring season during the cold exposure was higher than that of the other season because of the accumulation of the hair from the blood. Although the measurement of aldosterone cannot measure using both HPLC and ELISA in polar bear hair (Weisser et al., 2016), the aldosterone concentration could measure on the hair using ELISA in the present study. However, the summer season showed many samples were found to be below the measurement limit. The concentration rate used in the present study was 31.25 times. It is necessary to further increase the concentration rate. Because this rate is likely to change depending on species, age, and other factors, the concentration rate needs to be considered.

These results indicated the changes in both the hormone concentrations (cortisol and aldosterone) in the hair over a long period. This result suggested the changes were affected by the hair region factor, the hair color factor, and the changing meteorological conditions. However, there are many unknowns about the corticoids in the hair. Therefore, further researches will be required.

## CONFLICT OF INTEREST STATEMENT

The authors declare no conflicts of interest.
